# Donor specific HLA antibody in hematopoietic stem cell transplantation: Implications for donor selection

**DOI:** 10.3389/fimmu.2022.916200

**Published:** 2022-08-05

**Authors:** Scott M. Krummey, Alison J. Gareau

**Affiliations:** Immunogenetics Laboratory, Division of Transfusion Medicine, Department of Pathology, Johns Hopkins School of Medicine, Baltimore, MD, United States

**Keywords:** hematopoietic (stem) cell transplantation, HLA, donor-specific alloantibody, desensitization, haploidentical stem cell transplant

## Abstract

Advances in hematopoietic stem cell transplant (HSCT) have led to changes in the approach to donor selection. Many of these new approaches result in greater HLA loci mismatching, either through the selection of haploidentical donors or permissive HLA mismatches. Although these approaches increase the potential of transplant for many patients by expanding the number of acceptable donor HLA genotypes, they add the potential barrier of donor-specific HLA antibodies (DSA). DSA presents a unique challenge in HSCT, as it can limit engraftment and lead to graft failure. However, transient reduction of HLA antibodies through desensitization treatments can limit the risk of graft failure and facilitate engraftment. Thus, the consideration of DSA in donor selection and the management of DSA prior to transplant are playing an increasingly important role in HSCT. In this review, we will discuss studies addressing the role of HLA antibodies in HSCT, the reported impact of desensitization on DSA levels, and the implications for selecting donors for patients with DSA. We found that there is a clear consensus that moderate strength DSA should be avoided, while desensitization strategies are reported to be effective in most cases at reducing DSA to amenable levels. There is limited information regarding the impact of specific characteristics of DSA, such as HLA loci or overall level of sensitization, which could further aid in donor selection for sensitized HSCT candidates.

## Introduction

Hematopoietic stem cell transplantation (HSCT) is a life-saving therapeutic option for a wide range of malignant and non-malignant hematologic disorders. Historically, bone marrow transplant was performed on human leukocyte antigen (HLA) matched related or unrelated donors, due to the significant morbidity and mortality of graft-versus-host-disease (GVHD) associated with HLA mismatch (1–3). Advances in conditioning regimens, graft-versus-host prophylaxis, and HLA genotyping technology instigated new approaches for donor selection in order to increase the successful identification of donors and improve post-transplant outcomes. In cases where an HLA identical related donor is not an option, either haploidentical related or mismatched unrelated donors are often available.

In recent years, advances in HLA genotyping and clinical management have led to the successful use of mismatched donors in the form of haploidentical related transplant and permissively HLA mismatched transplants (3). While haploidentical HSCT has gained traction in recent years due to advances in the approach to manage engraftment and mitigate intense alloreactivity in both the graft and host directions. In addition, recent work has elucidated situations where mismatches of specific HLA loci carry a reduced risk of graft failure or GVHD. These “permissive” mismatches have been described based on the HLA-DPB1 allele and HLA-B leader sequence dimorphisms.

These advances have expanded access to transplant for many patients, particularly those from non-Caucasian ethnic groups where matched unrelated donors are less abundant (4). However, both HLA haploidentical and mismatched donors introduce the complication of donor-specific HLA antibodies (DSA), which can be a barrier to transplantation. Thus, increasing the use of HLA-mismatched donors in HSCT places increased importance on overcoming the barrier of DSA in HSCT. In contrast to solid organ transplant, HLA antibodies in HSCT represent a unique challenge in that the source of those antibodies, the host immune system, is replaced by the process of transplantation. Thus, if the deleterious effect of HLA antibodies on donor cell engraftment can be transiently reduced, there is a significant chance of long-term success. This has led to the routine use desensitization in HSCT programs using HLA mismatched donor-recipient pairing in the setting of DSA.

In this article, we will review the role of DSA in HSCT, the efficacy of desensitization approaches in mitigating DSA. From the perspective of HLA, we will discuss the conclusions and knowledge gaps that exist when considering DSA against potential HSCT donors.

## The growing complexity of HLA mismatched donors in HSCT

Among unrelated HSCT donors, multiple seminal studies established that minimizing the number of HLA mismatches is advantageous for survival and GVHD mitigation (5). Expert CIBMTR guidelines recently codified that there is no advantage to mismatch at any specific locus among HLA-A, -B, -C, or DRB1 (5, 6). Elegant work by Fernandez-Vina and colleagues found that mismatching at the low expression loci HLA-DRB3/4/5, -DQB1, and -DPB1 was associated with worse overall survival and transplant-related mortality among 7/8 matched unrelated cases, and had little impact among 8/8 matched cases (7). Thus, for unrelated mismatched HSCT, there is little consensus about the preference of HLA loci mismatch.

Recently, two bodies of work have begun to establish a hierarchy of outcomes related to HLA mismatch. HLA locus-specific algorithms have been identified to identify so-called permissive mismatches for HLA-DPB1 and HLA-B that do not carry an increased risk of GVHD or graft failure. HLA-DPB1 has historically not been included in HSCT genotyping and matching consideration. With advances in genotyping technology, many centers have access to HLA-DPB1 genotyping. Using an elegant series of *in vitro* experiments and bioinformatics, Zino and colleagues classified HLA-DPB1 alleles into groups based on the presence of alloreactive T cell epitopes, leading to the algorithmic assessment of permissive and non-permissive HLA-DPB1 mismatching between donors and recipients (8). Non-permissive HLA-DPB1 mismatching has been shown to be associated with worse outcomes, primarily non-relapse mortality and rates of GVHD (6, 9).

More recently, studies have uncovered the importance of HLA-B exon 1 sequence matching in HSCT outcomes. The HLA-B exon 1 encodes a nonamer peptide leader sequence that can be presented by HLA-E, which binds to T and NK cell receptors. The HLA-B leader sequence position two, unlike HLA-A and -C, is dimorphic, encoding either a methionine (M) or a threonine (T) (10). Recent work has evaluated the patient genotype and B leader sequence mismatch on GVHD and transplant outcomes. The International Histocompatibility Working Group evaluated nearly 34,000 unrelated HSCTs and found that among those with a single HLA-B locus mismatch, HLA-B leader mismatching was associated with greater GVHD risk versus leader matching, and that a shared T leader haplotype was lower risk than a shared M leader haplotype (11). Another study of unrelated HSCT cases with one mismatch at HLA-A, -B, -C, -DRB1, or -DQB1, found associations between HLA-B leader mismatch and genotype with mismatches at either HLA-DQB1 or -DRB1 that were associated with increased non-relapse mortality and GVHD (12). Thus, for unrelated mismatched donors, identifying the permissiveness of HLA-B leader mismatches is emerging as a way to improve outcomes in HLA mismatched unrelated HSCT. For unrelated donors, while the 10/10 HLA matching is ideal, this recent work has elucidated a hierarchy of mismatches that can lower the risk of GVHD and mortality.

The use of related HLA haploidentical transplantation has gained traction in recent years because of the significant expansion of the potential donor pool. While only 20-30% of patients have a potential HLA matched sibling, nearly all patients have haploidentical family members that could serve as donors (4). Multiple approaches are currently used to successfully manage the alloreactivity associated with haploidentical HSCT that result in high rates of engraftment and limited GVHD (4, 13, 14). Our center has reported successful outcomes using post-transplant cyclophosphamide in haploidentical HSCT versus matched related donors (15, 16). For these reasons, along with the speed at which donors can be evaluated for transplant, the use of haploidentical donors has been rising worldwide and represents another area in which HLA mismatch is increasingly being confronted (17).

Together, these developments have established new algorithms for HSCT donor selection. Transplant of mismatched donors by either approach, however, increases the likelihood of DSA complicating donor selection.

## HLA antibodies in HSCT

The HLA genes are the most polymorphic in the human genome, which have evolved to allow the immune system to sense foreign pathogens and provide wide-ranging protection (18, 19). The classical HLA genes are comprised of the Class I and Class II genes that present peptide antigen to T cells. To date, over 30,000 Class I and Class II alleles have been identified. The Class I HLA-A, -B, and -C genes are expressed on all nucleated cells, while the Class II HLA-DR, -DQ, and -DP genes are expressed on antigen presenting cells. Expression of Class II can be induced on other cell types, particularly in the setting of inflammation. Historically, HLA matching for HSCT patients and donors was performed for HLA-A, -B, -C, and -DR (8/8 loci matching), owing to limited typing methods that precluded typing at HLA-DQ and -DP (19). Currently, with the widespread and growing use of next-generation sequencing to perform genotyping, matching is clinically assessed at HLA-A, -B, -C, -DR, -DQ (10/10 loci matching) although data is available for donors and recipients at all HLA loci, including HLA-DP, -DRB345, -DQA1, and -DPA1.

Due to the high degree of polymorphism and the necessity of transplanting across HLA antigen mismatches in some cases, the presence of HLA antibodies directed against mismatched donor antigens has been recognized as a significant barrier to graft acceptance (20, 21). For HSCT patients, HLA antibodies can form through multiple mechanisms that activate HLA-specific humoral immunity. Major avenues include pregnancy, blood transfusion, and prior organ transplant. Blood transfusions are a common source of sensitization for patients undergoing HSCT, due to the effects of chemotherapeutic treatments for hematologic malignancy. In addition, inflammatory events such as vaccination, infection, or trauma can result in the formation of HLA antibodies, potentially due to cross-reactivity or bystander activation (22). Studies have estimated that approximately half of transplant candidates are positive for HLA antibody (20, 23, 24). Among HSCT candidates, multiparous females are more likely to be HLA sensitized than nulliparous females or males (20, 23).

## HLA antibodies as a barrier to successful HSCT

### Identification of DSA

Testing of patient sera prior to incompletely HLA-matched transplantation is critical to detect DSA that can impact engraftment, and to characterize the breadth and strength of DSA. DSA detection with solid-phase and cell-based assays is routinely performed in most HLA laboratories to inform clinical practice and stratify immune risk for patients, for both solid organ and bone marrow transplant programs (**Table 1**). Solid phase assays have been pioneered through the linkage of purified recombinant class I and II protein to microparticles labeled with fluorescent markers (25). Single-antigen bead (SAB) assays allow for the detection of DSA against each potential HLA mismatch, and the level of DSA in patient serum can be interpreted in a semi-quantitative manner using mean fluorescent intensity (MFI) values detected with Luminex-based technology (23, 26–28). However, due to the high sensitivity of this assay and the potential for false-positive reactions, cell-based crossmatch assays can be used to complement SAB results as they correlate with the ability of DSA to bind to donor cells *in vitro* (27). Traditionally, complement-dependent cell-based crossmatches were used to assess donor-recipient compatibility, but they have been largely replaced by crossmatches using flow cytometry because of the increased sensitivity. A flow crossmatch can provide valuable information in cases where patients have DSA that would be too low for detection by a complement-dependent crossmatch. Results from solid-phase and cell-based assays are invaluable in helping guide donor selection in the setting of mismatched HCT.

**Table 1:** Overview of methods used to detect donor-recipient compatibility and identify DSA.

HLA Antibody Detection Method	Features	Advantages	Disadvantages
Single-antigen bead (SAB) assay	Multiplexed detection of antibodies against ~100 HLA Class I and Class II antigens simultaneously.	• Most sensitive and granular method of identifying HLA antibodies. • Possible identification of allele-specific antibody.	• Potential detection of antibodies that are not clinically relevant. • False positive reaction patterns (e.g. denatured and cryptic epitope reactivity). • Variation in cut-offs for positive threshold between centers.
Flow cytometric crossmatch	Detection of antibodies that bind to donor HLA present on the cell surface.	Detects anti-donor HLA antibodies that are present at moderate strength.	• Reliability dependent on quality and source of target cells. • Difficulty in identifying HLA antibody specificities for highly and broadly sensitized patients. • Unreliable detection of low level but clinically relevant HLA antibodies.
CDC crossmatch	Detection of antibodies that bind to donor HLA present on the cell surface and can mediate cell lysis in the presence of complement.	Detects anti-donor HLA antibodies that are present at high strength.	• Not sensitive at detecting moderate or low-level HLA antibodies that are clinically relevant for HSCT.

### The impact of DSA in HSCT

The relationship between the presence of DSA prior to mismatched allogeneic HSCT and negative impact on primary engraftment and graft survival is now well-established (20, 29). While early studies evaluating the role of DSA in HSCT confirmed that a positive crossmatch was a risk factor for primary graft failure, a group of studies in the modern era of DSA detection using SAB results has provided more granular detail about the risk of high-level DSA in HSCT in various transplant settings (20, 21).

Among matched unrelated donor transplants, Ciurea et al. evaluated the impact of DSA in a cohort of 592 patients. Among the 19.6% of patients with HLA antibodies, eight had DSA against DPB1 (24). While the rate of primary graft failure was 3.2% among patients without DSA, three of eight patients with DSA had graft failure. In a retrospective case-control study of HSCT cohorts with or without failed transplants (groups of thirty-seven and seventy-eight patients, respectively), Spellman et al. found that 24% of the failed transplants had DSA against HLA-A, -B, or –DP, compared with 1% of the control group (30).

In umbilical cord blood transplants, Takanashi et al. found that the presence of DSA in twenty patients was associated with delayed time to neutrophil and platelet recovery (31). Among double unit umbilical cord blood transplants, Cutler et al. found that the presence of DSA was associated with an increased incidence of graft failure, prolonged time to neutrophil engraftment, and excess 100-day mortality or relapse (32).

Ciurea and colleagues at MD Anderson have conducted several seminal studies on the role of HLA antibodies and DSA in haploidentical HSCT. The 2009 study was the first to report a link between single antigen bead testing and primary graft failure in haploidentical HSCT patients (33). Among twenty-four consecutive transplant patients, the authors reported that three of four patients with DSA undergoing haploidentical HSCT failed to engraft, compared with one of twenty in a control cohort with no DSA. All patients with primary graft failure had one or more DSA with an MFI above 3,000. (Two of these patients were treated with TPE and rituximab, and one maintained DSA above 1,500 MFI).

A follow up study in 2015 evaluated 122 transplant recipients and found that twenty-two had DSA. Seven of eleven patients with DSA above 5,000 MFI failed to engraft (median DSA MFI 10,055), compared with none of the eleven with DSA below 5,000 MFI (median DSA MFI 2,471) (24). Five of the seven patients who experienced graft failure with DSA were also C1q positive. This study was central in establishing that DSA above threshold on single antigen bead testing carries a significant risk of graft failure. A recent study by this group evaluated the impact of desensitization of high-level DSA. The authors evaluated 37 patients who underwent desensitization for DSA versus a control cohort without DSA (n=345) (34). Patients with initial DSA >20,000 MFI and positive C1q after desensitization had lower engraftment rate, higher non-relapse mortality, and worse overall survival than controls. However, graft outcome and survival of patients with initial DSA <20,000 and those with negative C1q after desensitization were comparable with no DSA controls.

## Desensitization of HLA antibodies in HSCT patients

The removal of HLA antibodies in sensitized patients has been widely used for HSCT and solid organ transplantation. There are a number of modalities and protocols that are often used in various combinations. The major goals of therapies are to target B cells and plasma cells, physically remove antibodies from circulation, and to modulate the pathogenic functions of antibodies (21, 35). Here we will summarize the major therapeutic modalities and provide some context of how they are used in HSCT programs.

### Targeting B cells and plasma cells

Depletion of circulating B cells is one major component of desensitization strategies. The chimeric anti-CD20 monoclonal antibody Rituximab is the most widely-used agent, although several other agents are in development (36, 37). Targeted depletion of B cells with agents such as Rituximab has the advantage of not only physically removing alloreactive B cells, but also has tolerogenic effects, such as resetting the peripheral B cell pool away from a pro-inflammatory profile and altering the balance of the immunoregulatory plasma cell populations in the bone marrow and tissues (36).

Plasma cells are a major source of anti-HLA IgG. The proteasome inhibitor bortezomib, originally developed to treat multiple myeloma, has gained use in transplantation because it targets plasma cells. In pre-clinical models and kidney transplant patients, bortezomib has been shown to be effective in desensitization protocols in combination with other agents (38, 39). Additional proteasome inhibitory agents, such as carfilzomib, which has a reduced toxicity profile, are in development and being explored in the setting of HSCT (40).

### Physical removal of circulating DSA

Therapeutic plasma exchange (TPE) uses mechanical separation of the blood components to replace plasma with colloidal fluid. TPE has a wide range of indications and is generally well-tolerated. TPE is a mainstay approach to reduce the concentration of IgG in circulation during desensitization for several decades (41). In transplantation, TPE is also used to reduce antibody levels for solid organ transplant patients who are experiencing antibody-mediated rejection (41). There is no standard number of TPE procedures for desensitization, but typically patients undergo multiple rounds of plasmapheresis based on the levels of DSA as determined by the transplant center. Antibody levels are typically monitored with serial solid phase HLA antibody testing after treatments.

In addition to TPE, transfusion-based strategies have been employed to absorb circulating anti-HLA IgG. Two approaches are to use donor platelets or buffy coat infusion prior to transplantation to provide a target for the binding and clearance of anti-HLA antibodies (34, 41, 42).

### Modulation of antibody effector functions

Intravenous immunoglobulin (IVIG) is a preparation of polyclonal and polyspecific immunoglobulins. While initially developed to provide passive immunity in cases of immunodeficiency, high dose IVIG has immunosuppressive properties. High dose IVIG exerts several effects on immune cells *via* Fc receptors that attenuate innate and adaptive immune activation (41, 43). IVIG has been paired with TPE to prevent rebound of DSA by rapidly restoring the homeostatic levels of immunoglobulins in circulation. IVIG is used both for desensitization in solid organ and HSCT, as well as for treatment of antibody-mediated rejection after solid organ transplant.

## Efficacy of desensitization on HLA antibodies in HSCT patients

While a number of agents and strategies are used to desensitize patients prior to transplantation, these protocols and philosophies vary widely between transplant centers. There are both pre-transplant and post-transplant features that can be used to assess the efficacy of desensitization in HSCT patients (**Table 2**). While there are many larger studies that have reported the clinical outcomes of patients after desensitization, less information is available about the impact of treatment modalities on HLA antibodies and DSA. In addition, HLA antibody removal is frequently used in the setting of solid organ transplantation prior to transplant or for antibody-mediated rejection. Although there are caveats to extrapolating this information for HSCT patients (discussed below), there are many similarities between the two clinical scenarios. Here, we will review the available studies that have reported HLA antibody level information in the context of desensitization of both HSCT and solid organ transplant patients.

**Table 2:** Factors available to assess the efficacy of HLA antibody desensitization.

Pre-Transplant	Post-Transplant
Change in HLA antibodies • Solid-phase assay: antibody MFI, change in reactivity pattern, C1q status, titer • Flow cytometric crossmatch: B and T cell positive or negative	Engraftment • Days until chimerism (CD3+ and CD33+) Disease relapse Graft Versus Host Disease • Acute or chronic Clinical outcome • Non-relapse mortality • Opportunistic infection • Survival (overall and progression-free)

### Studies evaluating desensitization of HSCT patients

Leffell et al. evaluated fifteen HSCT patients with DSA who underwent desensitization (35). Based on levels of antibody corresponding to either a positive flow cytometric crossmatch (generally >10,000 MFI) or CDC crossmatch (generally >10,000 MFI at 1:8 serum dilution), patients underwent multiple rounds of desensitization with TPE, along with treatment with IVIG, tacrolimus, and mycophenolate mofetil. This cohort of patients each had from one to three DSA, with nine patients having Class I DSA, five possessing Class II DSA, and one patient having DSA against both Class I and II antigens. Three patients had DSA that were below the level of a positive flow cytometric crossmatch, eleven had DSA at a positive flow cytometric crossmatch level, and one was at a positive CDC crossmatch level. Those with DSA below flow crossmatch underwent one round of plasmapheresis, while flow crossmatch positive DSA patients received three to eleven rounds of TPE (average 5.6 TPE). After desensitization, the mean reduction for DSA was 64.4% (range 40.2-93.3%), with fifteen patients experiencing reduction in DSA levels. There was no reported association with the HLA specificity (loci or number of DSA) and the reduction in DSA. Three patients did experience a rebound in DSA level during the conditioning period. All fourteen patients who were transplanted engrafted by day 60; seven suffered relapses from 3-12 months after transplantation (29).

Bailen et al. evaluated nineteen haploidentical HSCT patients with DSA who underwent variable desensitization treatments with rituximab, IVIG, plasma exchange, incompatible platelets, and buffy coat treatments (44). Overall, twelve patients had DSA >5,000 MFI, six against Class I, 1 against Class II alone, and five against Class I and II. The mean reduction in MFI after desensitization was 74%, with a range from 20-100%. Ten of the twelve patients with pre-desensitization DSA >5,000 had DSA reduced below 5,000 MFI prior to transplant, although one patient had an increase in DSA levels prior to infusion to levels above 5,000 MFI (38).

A recent study by Ciurea et al. evaluated the impact of desensitization on thirty-seven patients at two institutions (34). Patients were treated with three sessions of plasma exchange followed by rituximab and high dose IVIG. Most patients received irradiated donor buffy coat as well. The mean DSA was 10,198 MFI, which was reduced to a mean of 5,937 after desensitization treatments. HLA antibody information was not reported by Class or loci. Patients were also evaluated for C1q positivity, which generally correlates with MFI. Fourteen of these patients had C1q positivity prior to treatment, and six became C1q negative after desensitization.

### Studies evaluating desensitization of solid organ transplant patients

Although there are multiple differences in the patient populations undergoing HSCT versus solid organ transplantation, desensitization of HLA antibodies has been used extensively in solid organ transplant with many of the same therapeutic modalities. Here, we highlight two detailed studies that have evaluated the impact of desensitization with plasmapheresis-based approaches on HLA antibodies.

Noble et al. evaluated the impact of desensitization in two cohorts of patients undergoing living donor kidney transplantation or deceased donor kidney transplantation and provided a careful evaluation of the impact of desensitization on HLA antibody MFI reduction (45). Patients were treated with rituximab, tacrolimus, mycophenolate mofetil, and steroids, as well as plasmapheresis protocols based on the strength of DSA. Seventeen living donor candidates were treated with plasmapheresis 4-5 times per week prior to transplant, and immunoadsorption was used if DSA was greater than 12,000 MFI. This cohort had a mean Class I DSA MFI of 6,195 and 2,191 for Class II. Twenty-seven deceased donor candidates with cPRA >80% underwent desensitization with the above agents plus multiple weeks of 4-5 sessions per week. This cohort had a mean Class I DSA MFI of 13,929 and 5,508 for Class II. For these patients, the mean decrease in MFI was 88% for Class I and 59% for Class II. Factors that were found to correlate with a greater decrease in MFI were the volume of treated plasma. Thus, these results demonstrate a similar reduction in Class I and Class II DSA by these desensitization protocols.

Yamada et al. reported the impact of desensitization in sixty-four renal transplant recipients who underwent TPE for AMR (46). Patients underwent approximately six procedures every other day followed by one dose of IVIG. HLA antibody testing by single antigen bead testing was reported at two time points, time point 1 after the third TPE, and time point 2, after the sixth TPE. Overall, the authors found that most of the reduction in DSA levels occurred over the first three procedures (time point 1), with smaller and more variable reductions occurring between time point 1 and time point 2. When compared with pre-treatment levels, Class I and Class II specificities had similar reductions at each time point, 25.7% and 25.1%, respectively, after the first set of procedures, and 37.1% and 34.2% after the second set. Among Class I loci, the largest reduction after all procedures was for HLA-A (48.6%) versus HLA-B and -C (27.2% and 32.2%, respectively). Among Class II specificities, HLA-DR, DQ, and DP had similar levels of reduction (39.8%, 33.6%, and 41.6%, respectively), while HLA-DR51-53 antibodies were only reduced by 19.9%. Interestingly, the authors found that for two specificities, the impact of treatments 4-6 (time point 1 to time point 2), HLA-DR51-53 and -DQ did not decrease, while all other loci did. Overall, this study provides a very detailed reporting of the impact of TPE desensitization on HLA antibodies by loci and number of procedures.

### Case reports and series reporting desensitization in HSCT patients

Several case studies have reported on HSCT in the face of high-level class I DSA in multiple donor-recipient scenarios, with most DSA directed at HLA-A and B antigens (**Table 3**). A patient undergoing evaluation for HCT had a 9/10 matched sibling donor with an HLA-A locus mismatch (patient A*32:01, donor A*24:02). The patient was highly sensitized, with A*24:02 DSA at 18,000 MFI. Desensitization with several TPE and IVIG treatments, plus two rituximab doses resulted in a decrease to an MFI of 2,233, predictive of a negative flow crossmatch (47). This treatment was also effective in reducing third-party MFI values. Spriewald and colleagues reported on a 53 year-old female patient with a CDC-level HLA-A2 DSA at 20,000 MFI that was treated with platelet infusions in combination with rituximab and bortezomib, reducing the HLA-A2 DSA to less than 5,000 MFI. This patient engrafted by day 10 with a 9/10 matched unrelated donor and remained in complete remission over 1 year post-transplant (48).

**Table 3:** Summary of DSA and desensitization methods used in HSCT and solid organ transplant studies.

Ref	Population	DSA Characteristics	Desensitization Modality
**HSCT Patient Studies**			
(35)	13 haplo, 2 10/10 matched UD	*DSA by HLA Class (no. patients):* 9 Class I only (5 FCXM positive) 5 Class II only (5 FCXM positive) 1 Class I and Class II (1 FCXM positive)	1-6 TPE and IVIG (possible 1-2 TPE and IVIG post-transplant) Tacrolimus and MMF 1-2 weeks prior to conditioning
(44)	20 haplo	*DSA by HLA Class (no. patients):* 10 Class I only (6 >5,000 MFI) 4 Class II only (1 >5,000 MFI) 5 Class I and Class II (5 >5,000 MFI)	Various including rituximab, IVIG, TPE, incompatible platelet transfusions, buffy coat transfusion, MMF, tacrolimus, steroids
(34)	37 haplo	*DSA by HLA Class (no. patients):* 14 Class I 12 Class II 11 Class I and Class II All DSA 10,198 mean MFI >20,000 MFI (6), 10-20,000 MFI (6), and <10,000 MFI (20)	3 TPE and IVIG, rituximab, buffy coat transfusion
**Solid Organ Transplant Patient Studies**			
(45)	45 renal transplant recipients (18 living, 27 deceased)	*DSA by HLA loci (frequency)*: HLA-A 77.7%, HLA-B 63%, HLA-C 15% HLA-DR 44%, HLA-DQ 36%, HLA-DP 28% *Living donor recipients:* Class I 6,195 mean MFI Class II 2,191 mean MFI *Deceased donors recipients:* Class I 13,929 mean MFI Class II 5,508 mean MFI	Various regimens of rituxumab, tacrolimus, MMF, steroids pre-transplant Apheresis (immunoadsorption, double-filtration plasmapheresis, plasma exchange) – multiple courses of 4 sessions Thymoglobulin and IV steroids on day 1-5 post-transplant
(46)	56 renal transplant recipients	*DSA by HLA loci (frequency)*: HLA-A 46.9%, HLA-B 35.9%, HLA-C 25% HLA-DR 48.4%, HLA-DR51-53 32.8%, HLA-DQ 54.7%, HLA-DP 15.6% 10 patients Class I only 22 patients Class II only 32 patients Class I and Class II	TPE and IVIG (mean 6.0 procedures)
**HSCT Case Reports**			
(43)	9/10 RD	HLA-A*24:02 18,000 MFI pre-transplant to 2,233 MFI day 0 FCXM positive to negative	20 sessions TPE and IVIG 2 doses rituximab (day -31, -9)
(41)	9/10 URD	HLA-A2 >20,000 MFI pre-transplant to 4,500 day -1 CDC positive to FCXM negative	7 platelet infusions with A2 donor 4 doses rituximab 4 doses bortezomib
(45)	haplo	HLA-B*35:01 14,142 MFI pre-transplant to 449 MFI day +14	Multiple platelet infusions with B35 donors 1 dose rituximab (day -8) 3 doses IVIG (days -7, -6, -5)
(46)	10/10 URD	HLA-DRB4*01:01 10,258 MFI to 8,525 MFI post-treatment DRB4*01:03 12,987 MFI to 10,702 MFI post-treatment	Plasmapheresis Rituximab

RD, related donor; URD, unrelated donor; haplo, haploidentical donor; FCXM flow cytometric crossmatch. Numbers indicate number of patients unless otherwise noted as frequency or MFI.

In cases where DSA testing is completed prior to the administration of blood products and not repeated prior to transplantation, new sensitization can present challenges and lead to graft failure. Following administration of eighteen platelet transfusions, a 58-year old male patient with no DSA detected previously developed high level DSA against his haploidentical donor (A*26:02, MFI 26,978; B*48:01, MFI 5,228) that was only detected post-transplant and resulted in graft failure and the necessity of a second salvage transplant (49). A similar case reported graft failure in a 64 year-old male on day 28 due to a *de novo* DSA against the donor’s A*11:01 (MFI 21,564) following haploidentical HSCT from the patient’s daughter (50). The only available donor for a second transplant was the patient’s brother, where a DSA to the donor’s B*35:01 (MFI 14,142) was also present. Desensitization with rituximab, IVIG, and multiple infusions with platelets bearing the donor’s HLA-B35 were successful in reducing the DSA to <500 MFI.

While less commonly found in the literature, cases reporting Class II DSA causing graft failure have also been published. DR53 antibody (DRB4*01:01, MFI 10,258; DRB4*01:03, MFI 12,987), detected pre-transplant in a patient undergoing transplant with a 10/10 matched unrelated donor. Although the DSA was reduced with plasmapheresis and rituximab, it was resistant to treatment and present at the time of transplant (DRB4*01:01, MFI 8,525; DRB4*01:03, MFI 10,702). This patient experienced secondary graft failure twice due to the HLA-DRB4 DSA, and a new donor with a DRB4*03:01N allele was used successfully (51). DPB1 DSA leading to graft failure following HCT from a 10/10 matched unrelated donor in a 53 year-old male has also been reported (52). In this case, the DPB1 mismatch (patient DPB1*04:01, 04:01; donor DPB1*01:01, 04:01) resulted in the development of DPB1*01:01 *de novo* DSA (MFI 13,752) leading to a strongly positive flow crossmatch that was predicted to be CDC positive as well based on the laboratory’s cutoff. This patient required a second HCT and the DPB1 DSA had a predictable impact on the donor search.

## Discussion

New approaches to HSCT have expanded the role of DSA evaluation in the process of donor selection. A strong body of work has established that HLA antibodies, and DSA particularly, is a significant barrier to successful HSCT. The major factor that has been shown to correlate with outcome is the strength of HLA antibody. Despite some ambiguity due to differences between techniques and laboratories, the overall consensus is that DSA above a value of 5,000 MFI, or at levels consistent with a positive flow cytometric crossmatch, is associated with worse outcomes for HSCT patients (24, 33). Additionally, based on a physical crossmatch-based assessment of compatibility, antibody at a level that would cause a CDC crossmatch is considered to be a contraindication to successful HCT. Thus, studies and case reports of desensitization mostly focus on antibodies that are above the flow cytometric crossmatch threshold of clinical relevance.

In other areas of histocompatibility and transplantation literature, there is evolving evidence of the importance of certain HLA loci mismatches and DSA formation over others. For example, multiple HSCT studies have evaluated the relative risk of HLA mismatches on survival and graft failure and failed to uncover an impact of mismatches at specific HLA loci (5). In solid organ transplantation, HLA-DRB1 and -DQB1 mismatches are associated with worse outcomes, and the degree of HLA-DRB1 and -DQB1 molecular mismatch load is associated with the development of post-transplant DSA (53–55). For the sake of choosing between donors in an HLA sensitized HSCT patients, the existing literature does not provide specific guidance beyond the importance of DSA strength. While some case studies have reported the specificities of DSA, there is little consensus about additional clinical or HLA-based factors that portent worse outcomes in HSCT patients with DSA. For example, we did not find any discernible evidence that Class I versus Class II DSA (or DSA against a specific loci) is associated with lower rates of engraftment or graft failure.

In the case of approaches to desensitization, existing studies provide evidence that reduction of DSA is a safe approach to transplant sensitized patients. In other words, the physical strength of the antibody at the time of transplant seems to be critical to the outcome of the transplant, not the characteristics of DSA prior to desensitization. Multiple studies have reported an association between successful transplantation and reduction in DSA strength of over 50% versus a starting level, with several reporting average MFI reductions in the 70-80% (34, 35, 44). This appears to be true across multiple desensitization protocols and institutions. Thus, for programs considering the outcome of desensitization against a given donor, the literature provides evidence to expect a reduction by over half of the starting level. However, as discussed below, there is relatively limited information provided about the impact HLA loci or other characteristics of the DSA on this outcome.

For example, at our center the presence and strength of DSA is a crucial factor that is considered when selecting among haploidentical donors (13, 56) Donors against whom DSA is present at high level (CDC crossmatch positive) are not considered further, while DSA at moderate levels (generally >3,000 MFI) are considered for desensitization. Desensitization consists of tacrolimus and mycophenolate mofetil along with a course of 3-6 IVIG and plasma exchange that depends on the strength of DSA (13, 20, 56). This regimen employs a wide range of treatment modalities and has been used successfully with minor variations for several years at our center.

### Limitations to the existing literature and goals for future studies

The current studies that evaluate the potential for desensitization have established that the presence of DSA above a moderate strength is predictive of a greater risk for graft failure. This work has provided important guidance for sensitized HSCT patients, and certainly has advanced the field for haploidentical and HLA mismatched transplants. From the perspective of DSA and histocompatibility, many of these studies have published limited HLA antibody data and overall there is little consensus on several areas related to histocompatibility. We find that the main factors that limit the strength of these findings and application to new HLA matching algorithms in HSCT:

*- Variable classification of antibody strength*. HLA laboratories use SAB testing from a limited number of vendors; however, there is variability in how assays are conducted, analyzed, and reported. As with most studies in histocompatibility, this makes the broad interpretation of HLA antibody levels (as defined by MFI) across different centers difficult.
*- Limited reporting of HLA loci in clinical studies*. The HLA system uses a complex nomenclature that makes interpretation difficult for non-HLA laboratory professionals. Many of the studies evaluated in this review provide very limited information regarding the HLA specificities and strength of DSA. This is probably due to a number of factors, but it precludes more detailed meta-analysis of the impact of HLA antibody characteristics in HSCT. Similarly, while the level of sensitization is frequently reported in studies of solid organ transplantation, most often as cPRA, this type of more global HLA antibody information is not included in HSCT studies. Again, while there is likely to be a relatively low level of HLA sensitization compared with solid organ transplantation and most studies would be underpowered to make strong conclusions, this data in aggregate could prove useful, particularly for multiparous females.
*- Limited statistical power to assess impact of DSA on clinical outcomes*. One inherent limitation in studying DSA in HSCT is the limited number of patients who both have DSA and experience graft failure. Even among the largest studies to date, the number of patients who experience graft failure with DSA is often less than a few dozen. Thus, no reliable conclusions can be drawn about loci-specific DSA (for example, is HLA-B DSA more associated with graft failure than DSA against other HLA loci? Is it more or less resistant to desensitization)?. As discussed above, more detailed reporting would potentially allow some idea of whether DSA directed against certain HLA loci are more problematic for engraftment or desensitization outcomes.

In future studies, it would be highly beneficial for more detailed HLA antibody information to be provided. Data in most studies evaluating desensitization is often limited to mean or median reductions in MFI among cohorts. More complete HLA antibody information might elucidate trends or patterns that are clinically useful. Examples of relevant data that could be included are: criteria for how HLA antibodies are defined at the study centers as well as more complete understanding of the sensitization status of patients, e.g. patient history, how many DSA are present, which HLA Classes, specific MFIs or MFI ranges, the presence of third-party antibody and cPRA classification.

It would also be informative to have more granular information regarding the impact of multiple low level DSA on outcomes. This would be valuable in the case of haploidentical transplantation, in which due to sensitization from pregnancy, multiparous female patients are more likely to have multiple DSA against related donors. It would be useful to know, for example, if there is a higher risk of transplanting across multiple DSA in this setting or if there is an increased risk of worse outcomes due to the relative durability of this mode of sensitization (e.g. the presence of high numbers of memory B cells). Fossey and colleagues provided some evidence that low level DSA in haploidentical transplant patients is not clinically detrimental. The authors reported on a group of five haploidentical HSCT recipients that were transplanted across borderline DSA (MFI between 1,700 and 4,200) with an overall survival of 80%, with only slightly longer neutrophil and platelet engraftment periods compared to patients with no DSA (57). It would be useful to expand upon this type of study, with reporting of the number and loci of DSA. However, there does not appear to be systematic studies of the impact of multiple low level DSA

As the use of HLA mismatched donors grows, however, more granular assessment of factors that impact desensitization for HSCT would be valuable. For example, when choosing between haploidentical donors with different DSA. In addition, when choosing HLA mismatched unrelated donors with DSA against HLA-B or HLA-DBP1, are there particular patient or HLA antibody characteristics that make desensitization more challenging? Assessment of loci-specific success of desensitization is difficult due to the range of HLA genes that precludes identification of cohorts with narrowly defined HLA antibody profiles. Demographic factors (sex, age) as well as immunologic/HLA factors (cPRA, etc.), could be considered.

In summary, the HSCT field is evolving in several key ways that make HLA mismatch and DSA an important consideration for donor selection. The existing literature has established that DSA can be a barrier to successful transplantation. In the future, the field will need to generate more specific information in order to continue to improve outcomes for patients undergoing HLA mismatched transplantation.

## Author contributions

SMK and AJG wrote and edited the manuscript. All authors contributed to the article and approved the submitted version.

## Funding

This work was supported by NIH R00 AI46271 (SMK).

## Conflict of interest

The authors declare that the research was conducted in the absence of any commercial or financial relationships that could be construed as a potential conflict of interest.

## Publisher’s note

All claims expressed in this article are solely those of the authors and do not necessarily represent those of their affiliated organizations, or those of the publisher, the editors and the reviewers. Any product that may be evaluated in this article, or claim that may be made by its manufacturer, is not guaranteed or endorsed by the publisher.
